# 137. Risk Factors of B-lactams Associated Cytopenias during Outpatient Parenteral Antimicrobial Therapy: Results from a Large National Sample

**DOI:** 10.1093/ofid/ofab466.137

**Published:** 2021-12-04

**Authors:** Yasir Hamad, Katelin B Nickel, Ige George, Yvonne J Burnett, Margaret A Olsen

**Affiliations:** 1 Washington University, St. Louis, Missouri; 2 Washington University in St. Louis, Saint Louis, Missouri; 3 University of Health Sciences and Pharmacy in St. Louis, St. Louis, Missouri

## Abstract

**Background:**

Cytopenias are rare complications of prolonged beta-lactam use; however, incidence and associated risk factors are not well described.

**Methods:**

Patients aged 18-64 years in the 2010-2016 IBM MarketScan Commercial Database discharged from the hospital on cephalosporin, penicillin, or carbapenem outpatient Parenteral Antimicrobial Therapy (OPAT) were included. The primary endpoint was hospital admission coded for neutropenia, leukopenia, or thrombocytopenia within the first 6 weeks post index discharge and within 7 days of beta-lactam discontinuation. Patients with history of malignancy and those who are on chemotherapy were excluded. Significant factors in univariate analysis were incorporated into a multivariable logistic regression model with sequential exclusion of variables with p > 0.1.

**Results:**

A total of 35,102 patients received beta-lactam OPAT; median age was 52 years and 53.6% were male. The primary outcome occurred in 150 (0.43%) patients at a median of 19 days (IQR 10-28 days after index discharge), which included 63 (0.18%) neutropenia, 85 (0.24%) thrombocytopenia, and 23 (0.07%) leukopenia admissions. Factors independently associated with readmission cytopenias included chronic liver disease (OR 4.61 [CI 2.93-7.25]), valvular heart disease (2.69 [1.71-4.24]), receipt of vancomycin (2.10 [1.42-3.12]), or antifungal therapy (4.42 [2.01-9.68]); lower risk was associated with carbapenem therapy (0.49 [0.32-0.75]) and diabetes (0.48 [0.31-0.74]) (Table 1).

**Conclusion:**

Readmissions with cytopenias during beta-lactam OPAT were rare and carbapenem use was associated with lower risk compared to other classes of beta-lactams. Combination of beta-lactam with vancomycin was associated with an increased risk of cytopenias, and those patients might benefit from closer monitoring.

Table 1. Factors Associated with Cytopenias during Beta-Lactams Outpatient Parenteral Antimicrobial Therapy (OPAT)

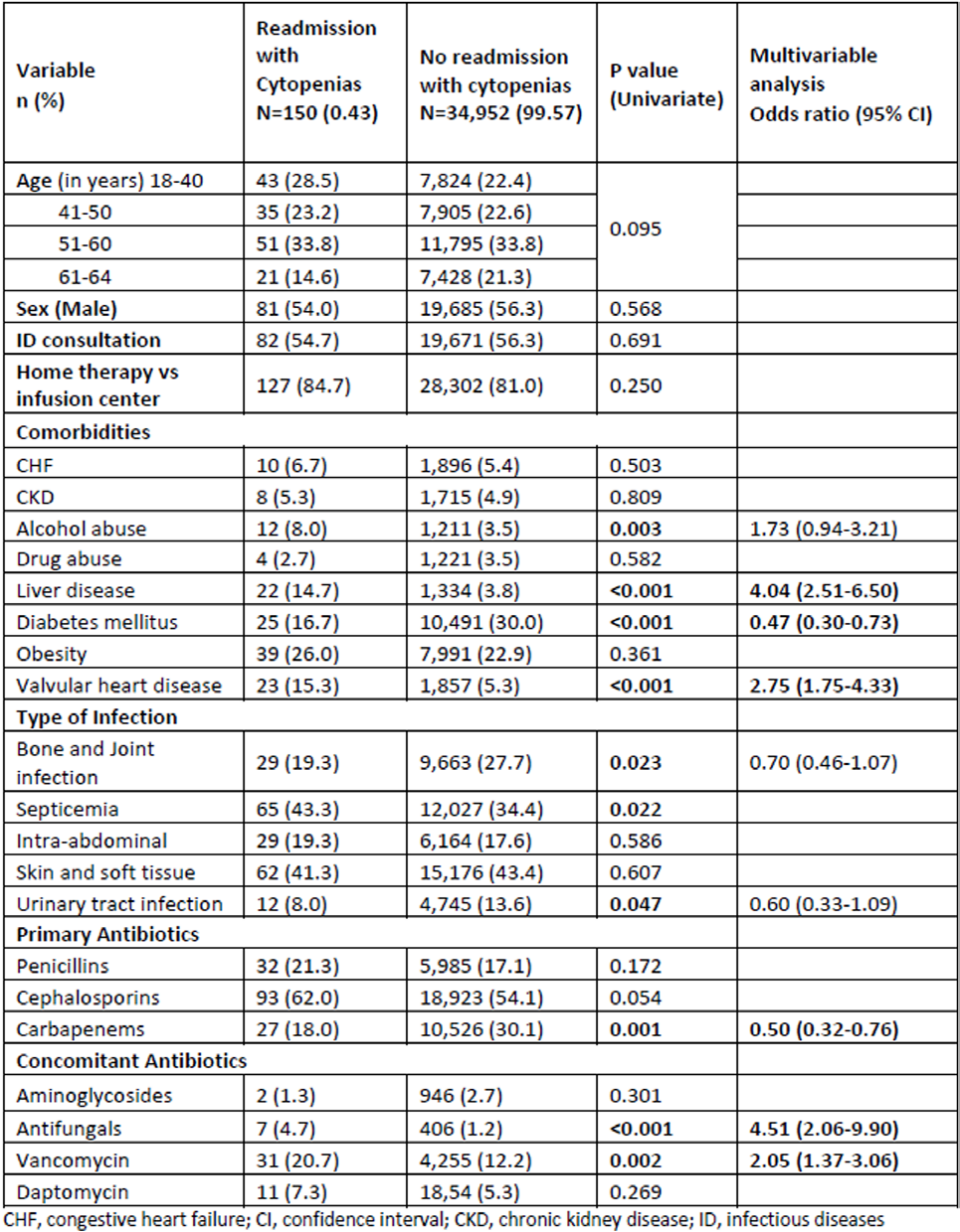

**Disclosures:**

**Margaret A. Olsen, PhD, MPH**, **Pfizer** (Consultant, Research Grant or Support)

